# Cross-sectional geometry predicts failure location in maize stalks

**DOI:** 10.1186/s13007-022-00887-x

**Published:** 2022-04-27

**Authors:** Christopher J. Stubbs, Christopher S. McMahan, Kaitlin Tabaracci, Bharath Kunduru, Rajandeep S. Sekhon, Daniel J. Robertson

**Affiliations:** 1 Department of Mechanical Engineering, University of Idaho, Moscow, ID USA; 2 School of Computer Sciences and Engineering, Fairleigh Dickinson University, Teaneck, NJ USA; 3 School of Mathematical and Statistical Sciences, Clemson University, Clemson, SC USA; 4 Department of Genetics and Biochemistry, Clemson University, Clemson, SC USA

**Keywords:** Bending, Biomass, Efficiency, Maize, Phenotyping, Lodging, Stalk, Strength, Stress, Structure

## Abstract

**Background:**

Stalk lodging (breaking of agricultural plant stalks prior to harvest) is a multi-billion dollar a year problem. Stalk lodging occurs when high winds induce bending moments in the stalk which exceed the bending strength of the plant. Previous biomechanical models of plant stalks have investigated the effect of cross-sectional morphology on stalk lodging resistance (e.g., diameter and rind thickness). However, it is unclear if the location of stalk failure along the length of stem is determined by morphological or compositional factors. It is also unclear if the crops are structurally optimized, i.e., if the plants allocate structural biomass to create uniform and minimal bending stresses in the plant tissues. The purpose of this paper is twofold: (1) to investigate the relationship between bending stress and failure location of maize stalks, and (2) to investigate the potential of phenotyping for internode-level bending stresses to assess lodging resistance.

**Results:**

868 maize specimens representing 16 maize hybrids were successfully tested in bending to failure. Internode morphology was measured, and bending stresses were calculated. It was found that bending stress is highly and positively associated with failure location. A user-friendly computational tool is presented to help plant breeders in phenotyping for internode-level bending stress. Phenotyping for internode-level bending stresses could potentially be used to breed for more biomechanically optimal stalks that are resistant to stalk lodging.

**Conclusions:**

Internode-level bending stress plays a potentially critical role in the structural integrity of plant stems. Equations and tools provided herein enable researchers to account for this phenotype, which has the potential to increase the bending strength of plants without increasing overall structural biomass.

**Supplementary Information:**

The online version contains supplementary material available at 10.1186/s13007-022-00887-x.

## Background

Yield losses due to stalk lodging (breakage of crop stems or stalks prior to harvest) are estimated to range from 5 to 20% annually [1, 2]. Stalk lodging, as opposed to root lodging [3], is defined as the loss of structural stability in the stalk, typically due to high winds [4–7]. As breeders select for higher performing varieties, it is important to be able to measure both the bending strength and the structural efficiency of the plant stem, ensuring that the structural biomass of the plant is located where it can provide the most benefit. In addition, it is important to identify the weakest section of the plant stem so that breeding efforts can focus on increasing stem strength only where it is needed. In other words, the lodging resistance or bending strength of any given plant is ultimately determined by the weakest internode (i.e., the weakest link). Reinforcing other parts of the plant by adding additional biomass will have no effect on stalk lodging resistance, if the weakest internode is not also reinforced.

Several methods of measuring stalk bending strength or other mechanical properties related to stalk lodging have been presented in the literature, including both laboratory based methodologies [1, 6, 8–17] and field based methodologies [18–21]. In addition, several studies have begun to examine the structural efficiency of plant stems [22], including correlating the strength of specific internodes to lodging rates in sorghum [23]. In this study, we investigate the morphology of mature maize stalks to (1) identify the weakest section of the stem, and (2) to determine the structural efficiency of the stem (i.e., how efficiently the plant allocates structural biomass). We also show that the morphologically weakest section of the stem is where structural failure is most likely to occur.

**Fig. 1:**
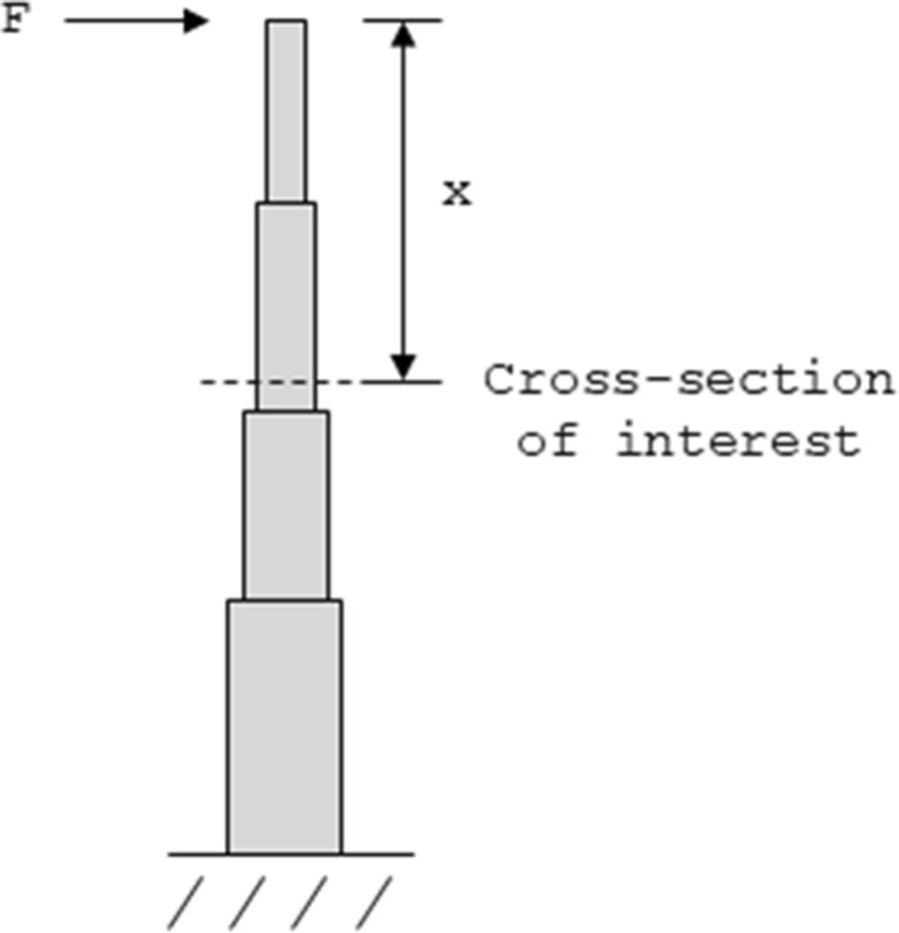
Loading diagram of a maize stalk, approximated as a stepped cantilever beam where F is an externally applied force (e.g., from the DARLING device) and x is the distance from the applied force to the cross-section of interest

A stalk lodges (i.e., breaks) when applied loads induce mechanical stresses that either cause the stalk tissues to fail (a.k.a. material failure) or buckle (a.k.a., structural failure) [9, 24–28]. Mechanical stresses in maize stalk can be calculated by approximating the stalk as a cantilever stepped beam with a transverse point load applied at the top of the beam [22, 29] (see Fig. 1). The maximum mechanical stress (σ) at any cross section in the stalk can be defined in terms of the externally applied load (F) the distance from the externally applied load to the cross-section of interest (x), and the section modulus (S) of the cross-section [30]:

1 $$\sigma = F\frac{x}{S}$$

In this study, an external load was applied to each stalk using a DARLING device [18], which applies a point load as shown in Fig. 1. This has previously been shown to produce accurate failure patterns in maize, and is representative of natural wind loading [22].

Section modulus is an engineering term that represents the morphology of a cross-section [7, 30]. Section modulus is used extensively to determine the bending strength and bending stiffness of engineered structures. Maize stalks are tapered, and the section modulus therefore increases as one moves from the apical internodes to the basal internodes.

From Eq. 1 and Fig. 1, we can observe that the location of maximum stress along the stalk is a function of both **x** and **S**. Importantly, the location of the maximum stress is independent of magnitude of the externally applied force. By measuring the morphology of the stalk one can predict the location of the highest stress, and therefore the location of failure.

## Methods

868 fully mature hybrid maize stem specimens (16 hybrids, 2 replicates, approximately 27 samples per plot) were subjected to: (1) in vivo bending tests to failure [18, 31], (2) morphological measurements of internode lengths, and (3) rind penetration tests to measure the cross-sectional morphology at each internode [11, 32, 33]. The morphological data were analyzed and used to predict the failure location of the stalks. The actual failure locations were then compared to the predicted failure locations.

### Plant materials

The 16 hybrids were developed by crossing B73 or Mo17 with a set of 8 genetically diverse inbred lines. These hybrids were developed to produce a broad range and stalk bending strength and flexural stiffness phenotypes. Table 1 displays the average bending strength and flexural rigidity of the stalks used in this study organized by hybrid. The hybrids were planted at Clemson University Calhoun Field Laboratory during summer 2019. The soil type in Clemson-CFL is Toccoa soil sandy loam to fine sandy loam. The hybrids were grown in a Randomized Complete Block Design with two replications. In each replication, each hybrid was planted in two-row plots with row length of 4.57 m and row-to-row distance of 0.76 m with a targeted planting density of 70,000 plant ha^−1^. The experiment was surrounded by non-experimental maize hybrids on all four sides to prevent any edge effects. To supplement nutrients, 56.7 kg ha^−1^ nitrogen, 86.2 kg ha^−1^ of phosphorus and 108.9 kg ha^−1^ potassium were added at the time of soil preparation, and additional 85 kg ha^−1^ nitrogen was applied 30 days after emergence. Standard agronomic practices were followed for crop management.

**Table 1:** Average bending strength and flexural rigidity of plant materials

Hybrid	Average bending moment at failure (Nm)	Average flexural stiffness (Nm^2)
PHZ51/Mo17	17.19	1.392 E−5
CH701-30/B73	25.24	3.191 E−5
CH701-30/Mo17	18.05	1.811 E−5
PHG35/B73	28.15	3.341 E−5
PHG35/Mo17	25.49	2.192 E−5
MoG/B73	38.73	7.136 E−5
PHPR5/B73	24.47	2.835 E−5
Mo45/Mo17	24.56	2.844 E−5
PHPR5/Mo17	28.14	3.206 E−5
Mo45/B73	28.22	4.807 E−5
MoG/Mo17	24.19	4.056 E−5
N7A/Mo17	25.31	3.126 E−5
A680/Mo17	26.85	3.785 E−5
A680/B73	19.94	2.771 E−5
PHZ51/B73	29.01	3.869 E−5
N7A/B73	27.85	3.688 E−5

### Bending tests & sample preparation

Stalks were submitted to in vivo bending tests to failure using the “Device for Assessing Resistance to Lodging In Grains” (aka DARLING) [18] at physiological maturity (38 to 42 days after anthesis). The DARLING device has been shown to produce the same failure patterns as naturally lodged stalks [18] and is highly predictive of the lodging propensity of different varieties [31]. Only healthy looking and intact plants were tested during our study. Any plants that were diseased or malformed (goose-necked, barren, mechanically damaged, etc.) were excluded from the study. Prior to testing, the tassel, leaf blades, and ear were removed, and the stalk was cut just above the ear bidding node. These steps were taken as our lab has found them to be “best practices”. In particular these preparatory steps mitigate oscillations in the stalk during testing and prevent interactions with neighboring plants. Oscillations and interactions with neighboring plants are two primary sources of experimental error in bending strength measurements. After preparing each stalk as outlined above the plant was loaded at the ear bidding node and deflected until failure.

After the in vivo bending test with the DARLING device, the stalks were cut at ground level and transferred to a greenhouse for drying. The greenhouse was maintained at ~ 38 degrees Celsius (100 degrees Fahrenheit) for 45 days at 35% RH to facilitate slow drying of the stalks and prevent the hollowing of the pith due to rapid drying. Stalks were dried to enable storage of samples without the tissues molding or rotting. Stalks were then stored in an air conditioned laboratory space until morphological measurements could be obtained.

### Morphology measurements

Internode lengths of each specimen were measured with a ruler. The location of stalk failure was also measured with a ruler. Other morphology measurements were taken at the midspan of each internode of every specimen. In particular, caliper measurements were used to obtain the minor and major diameters of each internode. Rind penetration tests were used to obtain the rind thickness of each internode and to calculate the Integrated Puncture Score [11, 32, 33]. Rind penetration tests were performed using an Instron universal testing machine. In particular, a probe was forced through the specimen at a rate of 25 mm/s, and the resulting force–displacement curve was analyzed using a custom MATLAB algorithm to calculate the rind thickness (t) of the stalk cross-section. The probe was 2 mm in diameter with 45 degree chamfer on its end such that the diameter at the tip of the probe was 1 mm. Additional details on the rind penetration test protocol are documented in [11, 32, 33].

### Predicting failure location

The failure location of each stalk was predicted using Eq. 1. In particular, this equation was used to calculate the bending stress (σ) at each internode. The internode which experienced the maximum bending stress on each stalk was predicted to be the internode which failed. The distance **x** was calculated by measuring the distance from the bottom of each internode of interest to the ear-bidding node, which is where the stalk was loaded during the in vivo bending test. The section modulus **S** was calculated by approximating the stalk cross section as a hollow ellipse. This method assumes that the rind is the primary load-bearing structure of the stem, and has previously been shown to be an accurate assumption [6, 7, 11]. The section modulus was therefore calculated using the minor diameter (d), major diameter (D), and rind thickness (t) [34].

2 $$S = \frac{\Pi }{32d}\left( {Dd^{3} - \left( {D - 2t} \right)\left( {d - 2t} \right)^{3} } \right)$$

### Data imputation

Due to the destructive nature of in vivo bending tests, direct measurements of some morphological characteristics were not obtainable at every internode. However, given the relatedness among morphological characteristics of the stalk we were able to infill these missing data via imputation techniques. In particular, we make use of the factorial analysis for mixed data (FAMD) approach [35] implemented by the *imputeFAMD* in the *missMDA* package in R [36–40]. In our implementation of this technique, we first aggregated all of the morphological characteristics (major diameter, minor diameter, and rind thickness) and integrated puncture score into a single data set; i.e., for each stalk we have a total of 4 features at each internode. We then imputed the missing values using the FAMD approach, with predictions being made based on 6 principle components; for further discussion see [35].

### Failure location prediction model

Once the imputation process was complete, internode specific stresses for the first six internodes were computed per Eq. 1 and Eq. 2. These stress values were max-normalized within each stalk. Proceeding in this fashion allows us to make comparisons across stalks; i.e., the feature under study herein is the proportion of the maximum stress that each internode experienced. The max normalized stress value was then paired with the failure status of the internodes, and a logistic regression model was fit, entering each of the max normalized stress predictors as a first order variable.

### Optimization measurements

In analyzing these stems, we can also pose the question: how much stronger could the stem be without increasing its total structural mass (i.e., how much stronger could the stem be if the structural biomass were more optimally distributed along the length of the stalk?). Said another way: what is the theoretical performance improvement of these stalks if one were to actively breed for uniformly distributed internodal stresses?

As can be observed in Eq. 1, as we move down the stem from the canopy to the ground, a larger external moment (**Fx**) is applied to each subsequent internode. Without any morphological differences in internodes, this would result in each basal internodes being more highly stressed than apical internodes, and failure would almost always occur at the bottom most internode. However, in practice, the cross-section of each stem increases as we move from the canopy to the ground to counteract the increased bending load. However, the change in cross-section (i.e. section modulus, **S**) is not always exactly proportional to the change in loading, and thus we see that some internodes are more highly stressed than others. Thus, there exists a theoretically optimized stem design, in which the section modulus and external loading vary at the same rate down the stem, resulting in every internode experiencing the same maximum stress. This theoretical stem morphology is considered optimized, meaning that reduction in structural biomass anywhere on the stem will result in a higher maximum stress.

**Fig. 2:**
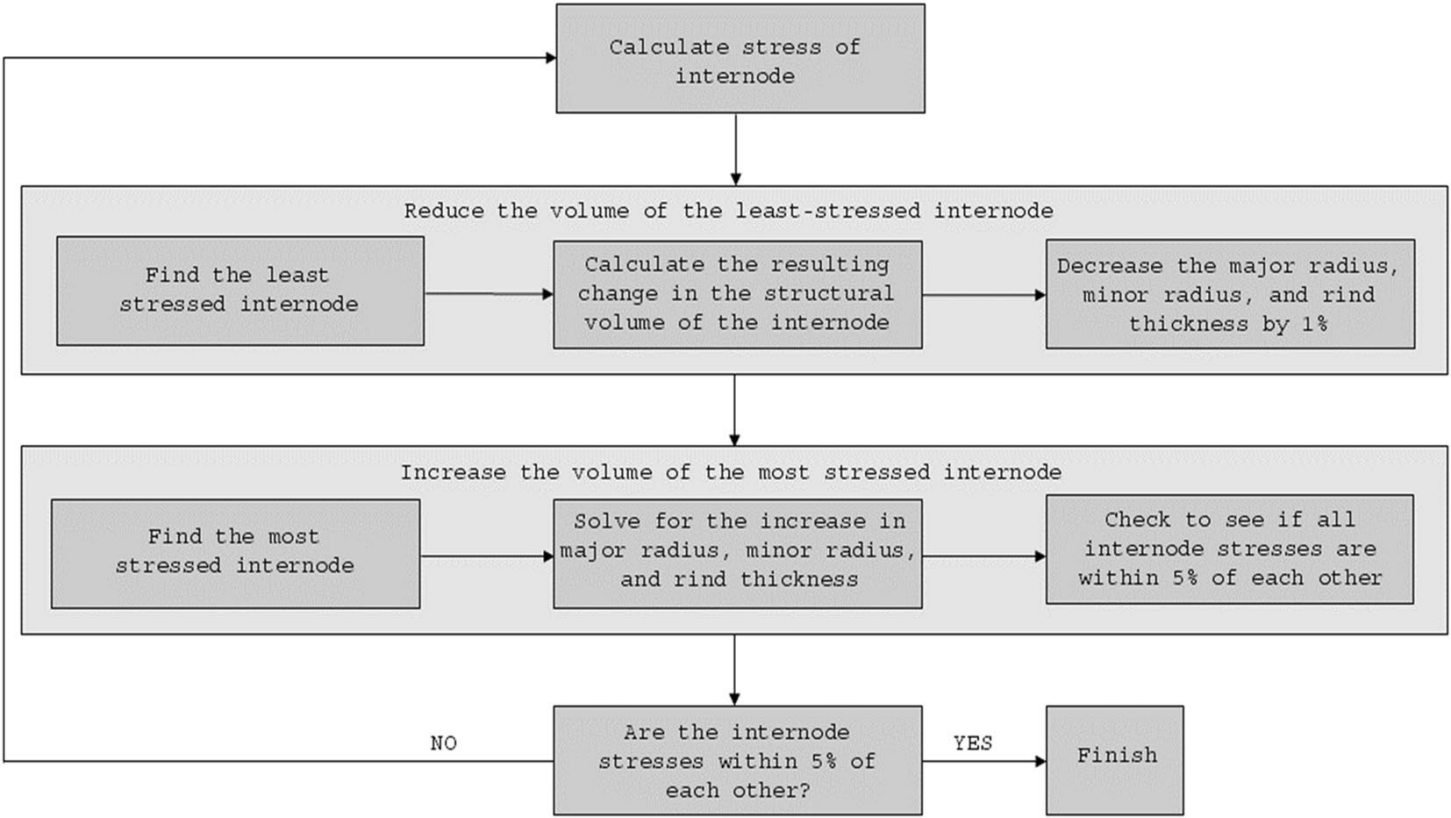
Block diagram illustrating the steps used in a custom optimization algorithm that computationally redistributed the structural biomass of stalks to achieve uniform mechanical stresses. As shown in the block diagram the algorithm iteratively/incrementally removed biomass from the least-stressed internodes and added biomass to most-stressed internodes until the solution converged (i.e., until all internodes’ stresses were within 5% of each other)

To investigate this, a custom optimization algorithm was developed in Matlab (MATLAB R2019a). For each stem, an iterative procedure was performed to re-distribute the structural mass between internodes until all the internodes were equally stressed. Figure 2 depicts a block diagram of the code, and the pseudo code is as follows:

1. Calculate the stresses of all internodes
2. Reduce the volume of the least-stressed internode
  - Find the least stressed internode.
  - Decrease the major radius, minor radius, and rind thickness by 1%. This ensures that the ratios between cross-sectional morphological properties remain consistent.
  - Calculate the resulting change in the structural volume of the internode.
3. Increase the volume of the most-stressed internode
  - Find the most stressed internode.
  - Solve for the increase in major radius, minor radius, and rind thickness that (1) increases the volume of the internode by the amount reduced in Step #2, and (2) maintains the ratios between cross-sectional morphological properties.
4. Check to see if all internode stresses are within 5% of each other. If not, iterate beginning at Step #2.

## Results

**Fig. 3:**
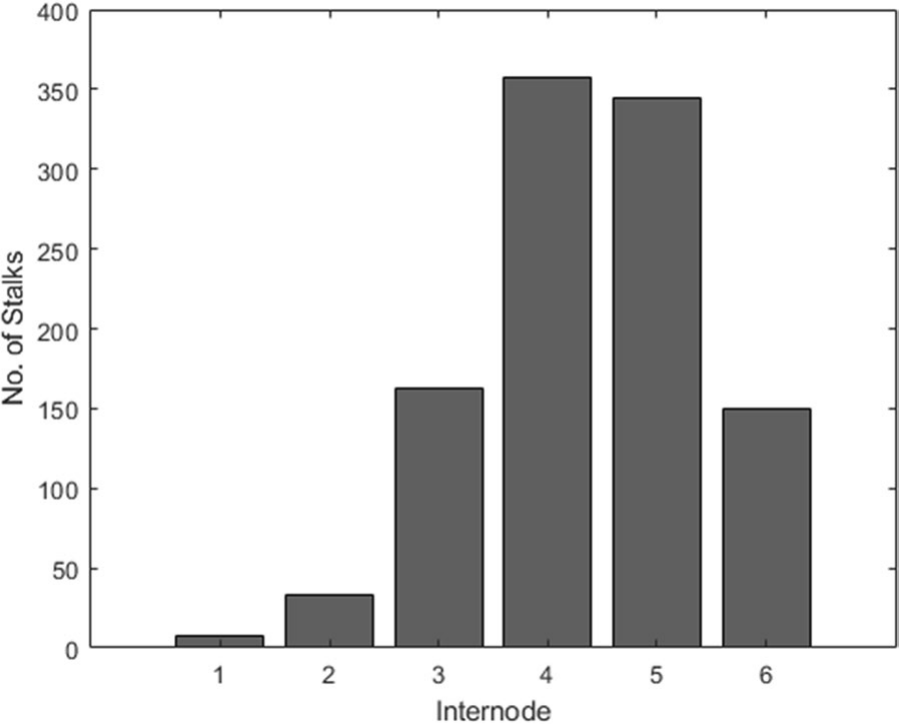
Histogram depicting which internodes failed (i.e., broke) during in vivo bending tests conducted with DARLING devices where internode 1 represents the internode immediately below the ear and internodes 2–6 are more basal internodes

Of the 868 maize stalks, 584 specimens failed at a single internode, while 284 specimens had fractures at multiple internodes. Figure 3 shows the distribution of the 1055 failed internodes for the 868 maize stalks where internode 1 is the internode below the primary ear bidding node and internodes 2–6 are more basal internodes. Cross-sectional morphology was measured at each internode of the stalks using calipers and rind penetration tests. Due to the destructive nature of the DARLING test, some internodes were damaged, therefore 3353 (16%) of the 20,832 morphological parameters measured in this study were imputed.

### Failure location prediction model

**Fig. 4:**
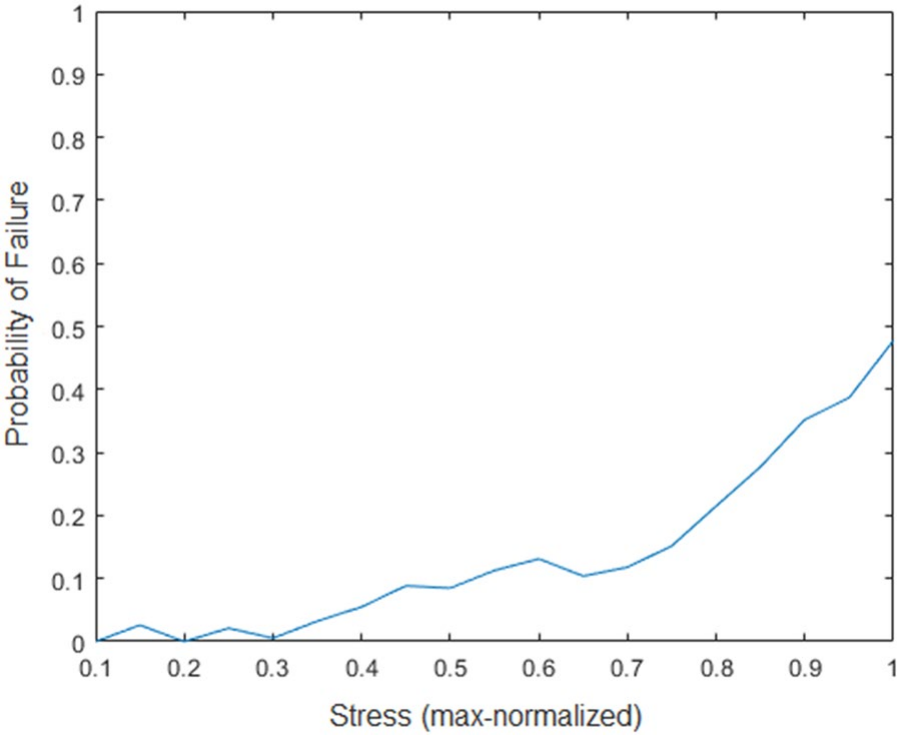
Line graph illustrating the probability of failure as a function of internode stress. As can bee see the in the graph the most mechanically stressed locations in the stalk are the most likely location at which failure will ultimately occur

Results demonstrated that internodal stress is highly related to failure location; i.e., the max normalized stress was significantly related to the likelihood of failure (p-value < 2e−16). The estimated regression coefficient was 2.83. This coefficient indicates that the likelihood of any particular internode failing increases as the max normalized stress of the internode increases; see Fig. 4.

### A computational tool for determining internodal stresses

**Fig. 5:**
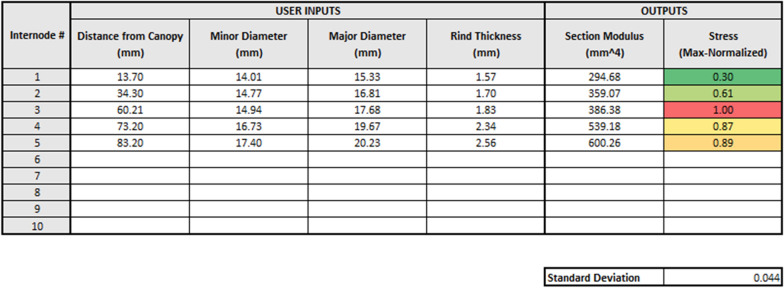
An example of the Excel spreadsheet that is provided in the Additional file 1, showing a stalk with five internodes. The user inputs morphological characteristics (viz. distance from canopy, major diameter, minor diameter, and rind thickness of each internode), and the spreadsheet will calculate the section modulus, max-normalized stress of each internode, and the standard deviation of the internodal stresses. The max-normalized stresses are automatically colored from red (maximum stress) to green (minimum stress)

To make calculation of internodal bending stresses more amenable to researchers without a structural engineering background (i.e., plant scientists, agronomists, and other end-users), an Excel (Microsoft Corporation, 2019) spreadsheet was developed, and is included in the Additional file 1. The user simply inputs the morphology of the plant stem, including the distance from the bottom of each internode to the top of the crop canopy and the cross-sectional dimensions of each internode. It should be noted that because the internodes typically fail at the meristem just above the node, the user can define the distance to the internode as approximately the distance to that internode’s basal node. Input values can be given for up to ten locations of interest along the length of the plant stem. The spreadsheet calculates the section modulus (S) and max-normalized stress at all locations. The max-normalized stresses are also calculated and automatically colored from red (maximum stress) to green (minimum stress). In addition, the standard deviation in the internodal stresses is calculated. This tool can be used by researchers to (1) calculate the most stressed internode and predict the location of failure for the stalk, and (2) determine the level of structural optimization of the stalk (e.g., the standard deviation of max normalized stresses). Figure 5 shows an example of the spreadsheet in which a stalk with five internodes is analyzed.

### Optimization measurements

**Fig. 6:**
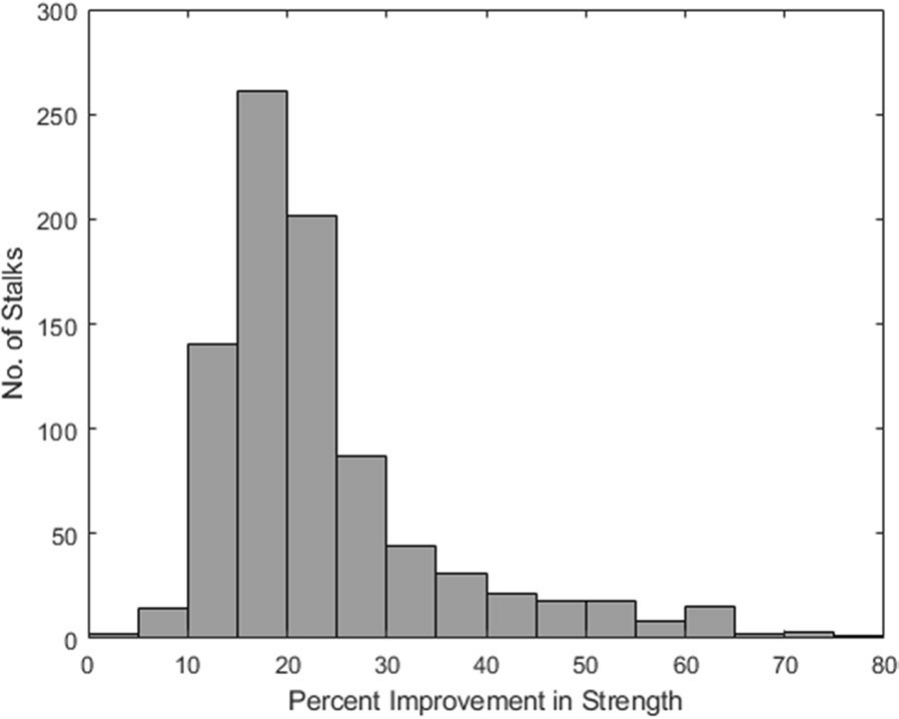
Histogram illustrating the theoretical improvement in stalk bending strength that could be achieved if the structural biomass of each of the 864 tested corn stalks was optimally distributed to create uniform mechanical stresses along the length of each stalk

The optimization algorithm described in Fig. 2 was performed on all 868 maize stalk specimens. In general, we found that significant improvement in bending strength could be achieved without increasing the total structural biomass of the maize stalks. Figure 5 depicts a histogram of percent improvement in strength that could theoretically be achieved without increasing total structural biomass for all of the stems investigated. The optimization process resulted in an average theoretical improvement of 23% in stalk bending strength, with many stalks experiencing over a 40% increase in stalk bending strength; see Fig. 6. This analysis represents the maximum expected performance improvement of these stalks that could be achieved without increasing structural biomass of the stalks.

## Discussion

### Predicting failure location

Results showed that stalk cross-sectional morphology was highly predictive of failure location. This demonstrates a clear link between stalk cross-sectional morphology (diameter and rind thickness) and stalk lodging resistance. A unique feature of this study is that the cross-sectional morphology was measured at multiple locations along the length of the stalk. The majority of previous studies investigating stalk lodging resistance have typically only measured cross-sectional features at a single internodal location (e.g., the basal internode or the internode below the primary ear bearing node). This is because the cost of measuring cross-sectional features of each internode is often cost prohibitive. The implied assumption in such research is that the stalk possesses a uniform taper. However, results from this study indicate that stalks do not possess a uniform taper and are therefore not uniformly stressed. Therefore, the cross-sectional features of a single internode may not be representative of the cross-sectional features of the entire stalk.

The nonuniform stress distribution in plants highlights the importance of loading plants in the most natural way possible when investigating stalk lodging resistance. While in vivo cantilever bending tests like the ones conducted in this study do not perfectly replicate wind forces they are a close approximation and are more economical than alternative in vivo testing scenarios (e.g., [41, 42]). Additionally, in vivo cantilever bending tests produce the same failure types and patterns observed in naturally lodged plants [18]. Laboratory based—long span three-point bending tests (e.g., [43]) also produce failure types that are similar to natural stalk lodging failure types. However, the stress distribution in a stalk is quite different in a three point bending test than in a stalk that has been loaded by the wind. In particular, failure will always occur near the loading anvil in a three-point bending test because this is where mechanical bending stresses are the highest. Because plant stalks are tapered, researchers utilizing the three-point bending methodology will usually place the loading anvil at the same anatomical location for every specimen in the study (e.g. the third node) [22]. In doing so the researcher has artificially induced the failure location (e.g., the failure will almost always occur just above the loaded node). The failure location chosen by the researcher may contain more structural biomass than is necessary relative to the rest of the stalk. This would cause an overestimation of the actual stalk lodging resistance of the plant and confound comparisons of bending strength between specimens. In nature, as well as during in vivo cantilever bending tests the failure location is determined by local material weakness and imperfections (i.e., suboptimal allocation of structural biomass). Thus in vivo cantilever bending tests are a better analog to natural stalk lodging resistance and should be employed when feasible. Short span three point bending tests of a single internode are highly problematic and should be avoided as described [43, 44].

### Optimization measurements

The lodging resistance or bending strength of any given plant is ultimately determined by the weakest internode (i.e., the weakest link). Thus, adding additional biomass or reinforcing any internode which is not the weakest internode will have no effect on lodging resistance. In other words, the idiotypic lodging resistant stalk would possess uniformly distributed bending stresses (uniform decrease in section modulus from the base of the plant to the top). Results showed that in general the stalks included in this study were not idyllic. In fact, we found that most stalks could theoretically be made as much as 23% stronger by changing the partitioning of resources during growth and development. In other words, if less structural biomass were allocated to the strongest internodes and more were allocated to the weakest internodes then the stalks would be more lodging resistant. This is a key finding because it suggests that it may be possible to significantly increase lodging resistance while minimally affecting harvest index and yield. Other methods of increasing bending strength and lodging resistance generally require the addition of structural biomass (e.g., increasing stalk diameter or rind thickness of the entire plant) which can potentially reduce yield as more resources are being used to strengthen the stalk making less available for filling the grain. In our previous studies, we have found remarkable variation in maize germplasm for partitioning of excess carbohydrates to various vegetative organs including stalks and cobs [45]. However, traditionally the stalk is treated as one large organ and characterization of natural variation for partitioning of resources to individual internodes and the impact thereof on overall strength of the stalks has been largely overlooked. Consideration of uniform internodal stresses and identification of genetic underpinnings of uniform allocation of resources across the stalks has the potential to increase the structural performance of the stalks without increasing structural biomass, as has been demonstrated and quantified in a previous study from our lab [22].

### Limitations

The DARLING has been shown to produce the same failure modes as naturally lodged stalks [18, 22]. This implies that a point force applied near the ear of a corn stalk is an adequate approximation of the types of forces stalks are subjected to in their natural environment. However, it is still not clear what the true loading profile of the stalks are in their natural environment. The loading profile has a significant role in the distribution of deflection and stresses along the plant, as the stress in Eq. 1 is modified to be *F x*^*n*^*/S*, where n is dependent on the loading profile. Some past studies have been performed which indicate that the true loading profile of windblown plants is similar to a point load (as is applied with the DARLING) [22]. The average wind velocity profile in crop canopies has been shown to be near zero in the bottom of the canopy and to rapidly increase in velocity near the top of the canopy [46] (likewise indicating that a point force may be a good approximation). However, further analysis is required to determine the exact drag force profile on the stalks. In reality the loading profile of the stalks in a field is continuously changing as a result of wind gust, eddy currents and interactions with neighboring plants.

This study was performed on dried hybrid maize stalks. As this experiment was focused on late-season lodging, it is not clear how these conclusions would extend to early season specimens (e.g. greensnap). In particular, the presence of a leaf sheath during plant growth and stem elongation can have substantial effect on bending stiffness and bending strength [47–49]. In addition, some of the results from the analyses could be different if inbreds (as opposed to hybrids) were studied. The authors have qualitatively observed that the collinearity between morphological characteristics does not appear to be as strong in inbreds as with hybrid varieties.

In this study, the stalk was assumed to be in pure bending, the structural contribution of the pith material was ignored, and the rind material was assumed to be homogeneous, isotropic, and linear elastic [50]. Material heterogeneity and anisotropy, or non-linear material properties would likely change the behavior of the analytical system. Specifically, the assumption of linear elastic material properties implies that the material is brittle. Although not entirely accurate, this assumption is considered valid for late season lodging experiments, where the material is senesced and dried. This assumption has previously been shown to hold when correlating stalk bending strength to historical lodging data [18, 31]. Further discussion of the influence of such material assumptions on equations has been investigated in a previous study by the authors [50]. These simplifying assumptions were deliberately made to allow for an easily-used generalized equation. Additionally, the equations used in this study assume small strains and small displacements. As such, these equations carry the same limitations as standard engineering beam bending equations, and may not be suitable to predict the stresses in the stalk with non-linear material behavior (e.g., greensnap).

## Conclusions

The presented experiments demonstrate that bending stress is predictive of failure location in maize stalks. As the bending stress is calculated through minimally invasive phenotyping, this means that failure location can be predicted without the need for destructive bending strength tests. A user-friendly computational tool was presented to help interested researchers phenotype for internodal stress. There remains significant room for improvement of structural mass allocation in maize stalks. Results are applicable to selective breeding and crop management studies seeking to reduce stalk lodging rates.

## Supplementary Information

**Additional file 1.** Internodal stress calculator.

## Data Availability

The datasets used and/or analyzed during the current study are available from the corresponding author on reasonable request.
